# Flow problems during implantation of a peritoneal dialysis catheter: building a capnoperitoneum using the peritoneal dialysis catheter

**DOI:** 10.1590/2175-8239-JBN-2023-0142en

**Published:** 2024-05-24

**Authors:** Elke Kathrin Franke, Ulrich Paul Hinkel, Christian Albert

**Affiliations:** 1 Central Clinic Bad Berka, Department of General Surgery and Visceral Surgery, Bad Berka, Germany.; 2 Central Clinic Bad Berka, Department of Nephrology, Bad Berka, Germany.; 3 University Clinic for Cardiology and Angiology, Medical Faculty, Otto-von-Guericke University, Magdeburg, Germany.

## Images in Nephrology

**Figure 1:**
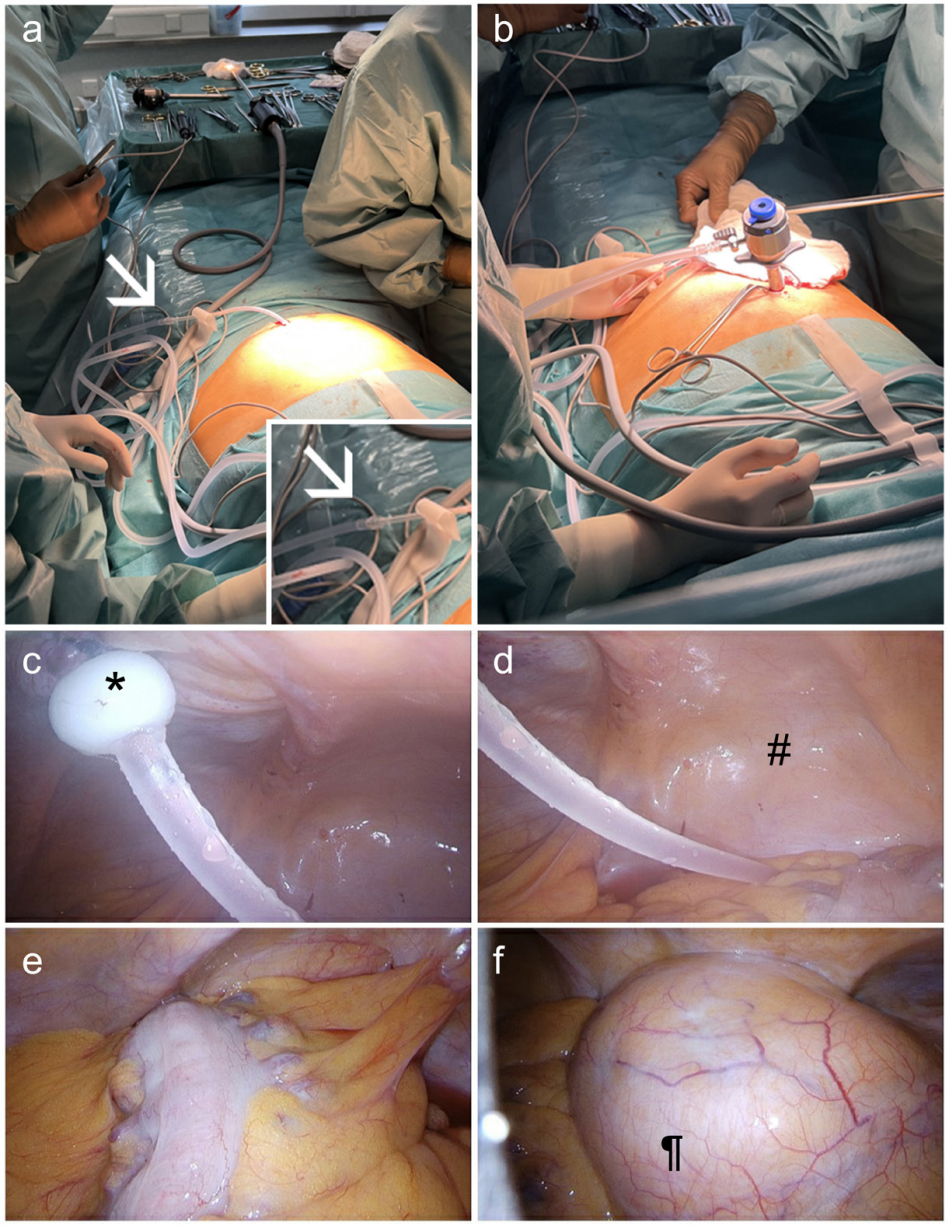
a. Carbon dioxide (CO_2_) inflation using the peritoneal dialysis catheter. b. Established capnoperitoneum with an inserted 10 mm trocar camera. c. For this patient, we used an Oreopoulos-Zellerman peritoneal dialysis catheter equipped with a flange and bead (*) surrounding the catheter below the inner cuff. The fascia transversalis is fixed with a purse-string suture between the flange and the bead to secure a tight sealing of the peritoneal cavity^3^. d. Bladder (#) and the peritoneal dialysis catheter entering the cavity of the small pelvis. e. Intra-abdominal adhesions adjacent to the colon in the lower left quadrant. f. Kidney transplant in the lower right abdomen (¶).

A peritoneal dialysis catheter (PDC) was implanted to initiate renal replacement therapy in an end-stage kidney-disease patient with chronic graft failure^1^. Before tunneling, the PDC was flushed with sodium chloride solution and aspirated without reflux. It was decided to explore the peritoneal cavity via laparoscopy^2^, which requires the artificial filling of the peritoneal cavity with carbon dioxide (CO_2_, capnoperitoneum), usually infused using a trocar with the risk of organ perforation or damage of the PDC during insertion. We found that connecting the CO_2_-gas line to the PDC allows the capnoperitoneum to be performed safely and easily (Figure 1). Afterwards, an incision is made in the epigastrium and a 10-mm trocar camera is inserted. On inspection of the abdominal cavity the entrance of the PDC appears to be unobtrusive and airtight using the suture technique by Twardowski^3^. The PDC entered into the small pelvis, without the need for an intervention^4^. After draining the capnoperitoneum, the PDC was rinsed again with a sodium chloride solution, which drained quickly and passively. Although no other catheter had to be implanted, it cannot be affirmed that the capnoperitoneum per se solved a suspected obstructive reflux problem.
